# Patients’ knowledge of diabetes foot complications and self-management practices in Ghana: A phenomenological study

**DOI:** 10.1371/journal.pone.0256417

**Published:** 2021-08-25

**Authors:** Irene Fosuhemaa Bossman, Shadrach Dare, Bright Anyimah Oduro, Prince Kyei Baffour, Thomas Kwadwo Hinneh, Jane Elizabeth Nally

**Affiliations:** 1 East Sussex NHS Trust, Eastbourne District General Hospital, Eastbourne, United Kingdom; 2 School of Health and Life Sciences, Glasgow Caledonian University, Glasgow, United Kingdom; 3 Mother and Infant Research Unit, University of Dundee, Dundee, United Kingdom; 4 Department of Vision Sciences, Glasgow Caledonian University, Glasgow, United Kingdom; 5 East Sussex NHS Trust, Conquest Hospital, St Leonards-on-Sea, United Kingdom; 6 Department of Public Health, Tain District Hospital, Nsawkaw, Ghana; Freelance Consultant, MYANMAR

## Abstract

**Background:**

The prevalence of diabetes is increasing in low and middle-income countries (LMICs) and over two-thirds of these are not diagnosed. Consequently, diabetes complications usually exist at the time of diagnosis. Foot ulcers is a leading cause of disability and mortality among diabetes patients.

**Purpose:**

To assess the knowledge and experiences of adult patients with Diabetes on diabetes complications and self-management practices with emphasis on foot care.

**Methodology:**

This applied phenomenological study design. Twenty patients attending Diabetes clinics were purposively sampled from two hospitals in Ghana. Face-to-face semi-structured interviews were conducted to evaluate patient’s understanding of diabetes and self-management practices. The interviews were audio-taped, transcribed, and analysed to generate themes using the constant comparison method.

**Results:**

Three-quarters of the participants in the study correctly defined diabetes as high blood glucose levels, but few knew the risk factors and complications of diabetes. Stroke and Hypertension were the most popular complications known, whiles diabetes foot complications were the least known. Almost all participants showed awareness of dietary self-management practices, but few had limited knowledge in foot care practices.

**Conclusion:**

Diabetes education in LMICs should promote self-management practices, especially foot care and clear dietary guidelines. There is also opportunity to invest in specialist diabetes training for healthcare providers and increase community-based care for people living with diabetes in Ghana.

## Introduction

The prevalence of diabetes worldwide has surged in the past two decades, and it is become a growing threat in low and middle-income countries. Currently, it is estimated that over 460 million adults globally live with diabetes, which is an increase by 62% from 285 million in 2009. This is estimated to rise further to about 578 million adults in 2030 [1].

Type 2 Diabetes is the commonest form of diabetes in low- and middle-income countries (LMICs) although other forms such as gestational diabetes and Type 1 diabetes are all prevalent [2]. With a growing prevalence of obesity, unhealthy diets such as fast foods, and increasing sedentary lifestyle and physical inactivity in developing countries, the burden of Type 2 diabetes is expected to increase further. The majority of the people living with diabetes in Africa, especially type 2 diabetes, however, remain undiagnosed [3]. In Ghana, for example, one in 52 adults in has diabetes and about 71.4% of these are undiagnosed [3]. As a consequence, diabetes complications such as neuropathy, renal failure, stroke, cardiovascular conditions and retinopathy usually exist at the time of diagnosis.

Diabetes related foot ulcers is a major contributor to disability. Up to 25% of people with diabetes develop a foot ulcer in their lifetime, which accounts for 85% of all lower limb amputations [4]. Limb amputation has a direct bearing on the individual’s value of life, and it is associated with high mortality and socio-economic cost [3]. The burden is even greater in poor and rural communities where there is little support for disabled people or populations that are engaged in manual jobs such as farming. To prevent this unwanted disability from occurring, IDF recommends foot assessment and foot care practices at least annually for people with diabetes [5]. Yet, in Ghana, scheduled diabetes foot assessment is rarely practised, though most patients with diabetes have been reported to have peripheral neuropathy [6].

Diabetes self-management (DSM) forms a significant part of diabetes management, and there is evidence that adults with diabetes who perform self-management activities experience better health outcomes and improved quality of life [7]. The main concepts of DSM behaviours that predict good health outcomes include: adherence to a dietary regime, physical activity, blood glucose monitoring, medication adherence, healthy coping skills and risk reduction behaviours such as foot care practices [8]. The ability to self-manage their condition depends on sociodemographic factors as well as clinical factors including complexity of treatment regime and co-morbidities. In addition, systemic factors such as social support and communication with health care providers play a critical role [9]. In a systematic review, Nam and colleagues [10] found that a lack of knowledge, negative attitude and beliefs about diabetes and its management, divergent cultural and spiritual values, social support and financial constraints hinder patients from managing their condition effectively. It is likely that these factors also undermine diabetes self-management in Africa [11].

The majority of literature on diabetes in Ghana have explored patients knowledge of diabetes, and very few studies have assessed patients’ knowledge of diabetes complications [12], dietary management [13], ocular manifestations [14] and explanatory models for type 2 diabetes [15]. To the best of our knowledge, none of these studies evaluated patient’s knowledge on foot care practices.

The aim of this study, therefore, is to evaluate the knowledge and experiences of adults with diabetes on diabetes complications and self- management practices with emphasis on foot care in two Ghanaian hospitals. The study findings will guide diabetes care providers to strengthen their support for patients, by designing effective educational sessions to boost self-management practices, prevent diabetic foot complications and enhance patients’ quality of life.

## Methodology

### Research design

This study adopted a phenomenological design to evaluate patient’s understanding of diabetes, its complications and their personal experiences with self-management practices. Face-to-face in-depth interviews (semi-structured) were conducted using tools designed by the researchers. A phenomenological approach was considered as suitable because it describes a phenomenon or concept from the subject’s own perspectives and experiences [16].

### Ethics approval

The study was approved by the Ethics Review Board at Glasgow Caledonian University, Glasgow (ref no: HLS/LS/A17/010). In Ghana, permission to conduct the study was granted by the Heads of the Institutions, upon the advice of the Hospitals’ Research and Development Committee, who ensured that the study protocol was ethically sound and the lead researchers were clinically qualified to conduct diabetes screening in Ghana [IFB, SD and BAO are registered clinicians in Ghana]. In addition, signed consents were obtained from all participants before participation. Prior to consenting, an information sheet which described what the study was about, participants’ rights to voluntary participation or withdrawal at any point during the study, and the risks and benefits for participation were explained to them. In addition, participants were assured of anonymity and confidentiality. There were no financial rewards for participation, however, the research team examined respondents’ feet and eyes after the interview for signs of diabetes complications and given necessary support, advise or referrals. Other non-participants also received care and advice regarding their disease management. We, therefore, do not have reason to believe that this may have influenced participation or even participant’s responses. These clearances satisfy the national guidelines to conduct research in the Ghanaian clinical setting. The researchers were, however, not considered foreign because most of the researchers are registered clinicians in Ghana.

### Study setting

The study was conducted in two public district hospitals in Ghana: Sampa and Mankranso Government Hospitals. Sampa is the largest community in the Jaman North district in the Bono Region of Ghana with a population of over 26,000. It is a largely urban area, with about 80% of the inhabitants engaged in agriculture and forestry. About 19 and 37 percent of the male and female populations are non-literates respectively [17]. At the time of the study, health care in the district was provided by one government hospital, two health centres and community health posts. Mankranso is the capital of the Ahafo-Ano South district in the Ashanti region of Ghana. About 28% of the people aged 11 years and above are illiterates and the predominant occupation is agricultural forestry and fisheries [17]. At the time of the study, healthcare in the district was provided by five clinics, nine health centres and three hospitals. The Mankranso government hospital is the largest.

In both districts, health centres (clinics) and community health posts are the first point of call for most patients, but specialist diabetes care is managed at the district hospitals. Patients attend these hospitals for prescriptions and blood investigations and general assessments, but some patients attend to the health centres to check their blood glucose levels. The district hospitals also serve as the referral centre for the surrounding rural health centres. These district hospitals were purposively selected, over health centres and community health posts, because of they offer specialist diabetes clinics and are the largest facilities in the districts.

### Sample recruitment

We purposively selected twenty adults (18+ years) who have been diagnosed with diabetes for at least the past six months and were attending specialist clinic at the selected district hospitals. Our aim was to interview patients who will have prior experience managing diabetes. A previous study suggested that 20 participants are sufficient to achieve saturation in qualitative studies [18]. Hence, for this study, we considered 20 patients [Mankroso (*n* = 9), Sampa (*n* = 11)] sufficient to provide rich and accurate data about patients’ knowledge and understanding of diabetes complications.

### Data collection

Data for this study were collected using a researcher-developed semi-structured face-to-face interview. The interview guide was designed via a review of relevant qualitative studies (For example, Doherty et al., [13] and deGraft et al [15] and a pre-identified conceptual framework: the self-regulation model (SRM) or the personal model. The model emphasises people’s representation of their illness, and how it directs them to engage in self-care behaviours to prevent disease complications [19]. The guide served as a checklist during the interview and also ensured that the same basic lines of inquiry were pursued systematically for each person interviewed.

The interview guide was piloted among ten patients attending a diabetes follow-up clinic at Dormaa East district hospital in the Bono region. This helped to assess the suitability of the study tools- whether they were understood by the respondents, and to familiarise ourselves with the preferred language translations and phraseology among the population. The patient interview guide was structured in English but was conducted in the Akan language, which is the local dialect of the study population, as the vast majority of the sample were not fluent in English.

The interview guide was in two parts; the first part recorded the patient’s demographic and vital statistics including age, sex, body mass index, blood pressure, fasting blood glucose, educational status, occupation, past and present medical history, duration of diabetes, previous foot ulcer and medication history. The variables age, sex, educational level, occupation, duration of diabetes and medical history were self-reported by the patients. Information on the patient’s fasting blood glucose level, body mass index and blood pressure recordings were retrieved from the patient’s latest medical records. The second part assessed the patient’s knowledge of diabetes complications, self-management practices, diabetes foot ulcers, foot care practices and experience in diabetes foot assessment. In addition, participants were encouraged to speak up in case they had anything further to say related to the topic, and we used follow-up questions to clarify unclear or ambiguous statements.

The interviews were conducted by IFB and supported by BAO and PKB. All have at least a master’s level qualification and clinical experiences in Diabetes management and undertaken courses in research methods. As a result, they were familiar with qualitative interviewing techniques, and conversant with the terminology in diabetes care. In addition, these researchers are Akan, and are proficient in the local language. After the interview, the transcripts were discussed among the research team which includes researchers with experience in qualitative research and clinical expertise. The interviews were conducted in 2017.

All interviews were conducted in private rooms in each facility to ensure privacy. The interview took place over two weeks; one week each at Sampa and then at Mankranso Government hospitals. We achieved saturation after about 16 interviews. The interview proceedings for each participant lasted for about 20–30 minutes, and were audio recorded with respondents’ verbal and written permission. In addition, short notes on patients’ comments were jotted in a journal during the interview to aid in reflection for transcription purposes. Following the interview, the research team assessed the feet and vision of all patients attending the diabetes clinic (both participants and non-participants of the study) for ulcers, risk of neuropathy, diabetes retinopathy and structural deformity in a one-to-one consultation. Based on patient’s risk status, they were educated and referred for appropriate treatment.

### Data analysis

Thematic analysis was used to analyse the interview data. Verbatim transcriptions for all recorded interviews were conducted in the Akan language, and subsequently, translated into English. All the transcripts were then read thoroughly to find recurring themes or excerpts that conveyed the relevant information. The researchers used hand coding to note key words and phrases in the transcript, which helped to identify sections for allocating initial codes. Although no special computer-assisted software for qualitative analysis was used for the coding, the “find and search” functions of the Microsoft Word office software helped to locate recurrent statements in the transcript. We developed emerging code statements from thoroughly reading patients’ responses in the transcript and compared to existing literature.

Using constant comparison, the significant code statements were grouped into broad themes supported by participants’ critical quotes. Data saturation was reached on most of the themes. In addition, embedded quotes approach was used to bring in the voice of participants in the study. These quotes provide specific real evidence in the participants’ words, to support the theme. The anonymity of participants was protected by masking their names in the data. Finally, the qualitative data files were organised, stored and backed up on a password protected computer for future reference, and also in accordance with our ethics approval.

## Results

### Characteristics of participants

Overall, 20 patients with Diabetes were selected in this study. The characteristics of the study participants are shown in Table 1. The majority of patients were women, had no formal education, were farmers and had been diagnosed with diabetes within five years of the study. Most of the participants had at least one comorbid condition and the commonest was Hypertension, but fewer had a history of foot ulcer and one had an amputation.

**Table 1:** Characteristics of participants.

Characteristics	Participants (N = 20)	Percentage (%)
**Sex**		
Female	15	75
Male	5	25
**Age (years)**		
≤ 49	4	20
50–59	5	25
60–69	7	35
≥70	4	20
**Education**		
No education	14	70
Basic education	6	30
**Occupation**		
No occupation	4	20
Farmer	12	60
Trader	3	15
Security guard	1	5
**Duration since diagnosis (years)**		
<1	1	5
1–5	12	60
6–10	4	20
>10	3	15
**Comorbidities**		
No other condition	4	20
Hypertension	14	70
Peptic ulcer disease	2	10
**Diabetes foot disease history**		
No history of ulceration	15	75
Previous ulceration	3	15
Active ulcer	1	5
Previous amputation	1	5

Thematic analysis of the interview transcripts identified four main themes: Knowledge of diabetes and its related complications; Beliefs and experiences about foot complications of diabetes; Diabetes self-management practices and Experience with diabetes foot assessment.

### Knowledge of diabetes and its related complications

Majority of the participants explained diabetes as a condition associated with high blood sugar, but knowledge about the risk factors and associated complications was limited. It was especially common for respondents to describe the symptoms of diabetes based on their individual symptoms- such as dizziness, dizziness, frequent urination and tremors. Although few (*n* = 5) participants recalled a family history of diabetes, they could not associate it as a risk factor.

*“Yes*, *I was aware of the diabetes condition because both parents had diabetes…*. *No*, *not at all*, *I did not know I will be at risk”* (60-year-old female farmer)

Most of the respondents were aware of stroke and hypertension as complications of diabetes, but only two of the participants cited foot ulcer and vision problems as possible complications of diabetes, and one participant did not know about any diabetes complications.

*“I know diabetes can lead to stroke*, *and one can develop an ulcer that will be difficult to manage and heal”* (48-year-old female respondent).

*“I am not sure I know of such complications…*, *it will result in death anyway”* (82-year-old farmer).

The main source of diabetes knowledge among the study participants was from attending diabetes clinic and participants placed high premium on the education they received from the diabetes clinic, but others based their knowledge of diabetes on their personal experiences.

*“Yes*, *the education is beneficial*, *and the educators seek to clarify any concerns anyone may have as well*.*”* (65-year-old male with 7 years duration of diabetes*)*

### Beliefs and experiences about diabetes foot complications

There were varied perspectives with regards to patients’ beliefs and experience of diabetes foot complications. The majority of respondents who had no previous history of foot ulcer were ignorant of the causes of diabetes foot ulcers, while others with a previous or an active foot ulcer were aware of diabetes foot complications. Also, patients with long duration of diabetes (more than 5 years) who experienced abnormal sensations/symptoms of the foot were particularly more alert of diabetic foot complications compared to their counterparts with short duration of diabetes (less than 2 years) and with no abnormal feet problems. Some respondents had knowledge of diabetic foot ulcer because an immediate relative with diabetes had a chronic diabetes foot ulcer.

*“… an aunt of mine died from a complicated foot ulcer*. *She had a sore on the foot which spread through to her bones and other parts of the body*.*”* (59-year-old female respondent with 18 years history of diabetes)

Only a few participants mentioned there was a connection between glycaemic control and foot complications. Some attributed the causes of diabetes foot ulcers to a noticeable open sore from an external injury. Although most of the participants reported symptoms like numbness, tingling and burning sensations, they did not know it could be due to diabetes, and so when the medications from their healthcare provider was not providing needed relief, they resorted to self-medication.

*“Yes*, *I learnt that if you have diabetes and develop a cut from anything sharp*, *it leads to diabetes foot problems*. *However*, *I suddenly saw this ulcer develop*. *I did not have any cut or anything which still baffles me*. *sometimes I feel sudden pains in my feet akin to having an electric shock*” (49-year-old female with less than a year history of diabetes)

*“I experience the pain only on my feet to the point that I find it difficult to bath the area*. *The pain emanates from the big toe*, *throughout to the plantar surface of my feet…*. *I don’t even know how it started that it has become darkened suddenly”* (80-year-old women).

Few patients also remarked that the slow healing rate of foot ulcers in diabetes was a major problem, but they did not understand why.

*“I have been told that when someone with diabetes develops an ulcer*, *it may become problematic if unattended to* (49-year-old with less than a year history of diabetes)”

Despite the lack of a better understanding about diabetes foot complications, slightly over half of the subjects knew that lower limb amputations could occur in people with diabetes. Their knowledge stemmed from direct personal experiences or indirect involvement with victims of diabetes-related lower limb amputations.

*“No*, *I didn’t know [*that the ulcer could lead to amputation*]*. *But the amputated foot developed from a stone cut*. *I immediately reported the incident to my mum who asked me what happened*, *and I narrated that I had a wound beneath my foot*. *About three days after the cut*, *I was not able to do anything and felt weak*, *hence my brother brought me to the facility*. *My dad has passed on*, *but anytime I asked them to take me to the hospital regularly*, *they felt reluctant because they had no money to do so”* (64-year-old farmer)

### Diabetes self-management practices

Overall, participants exhibited general knowledge on the day-to day activities they undertake for their diabetes management. The main aspects of their management mentioned included: dietary practices, exercises, medicines adherence and foot care practices. Most of the participants were confident they had received enough education on dietary management of diabetes. However, more than half of the participants reported having received very limited education with regards to foot care practices.

#### Dietary practices

In all cases, patients referred to diet as the core component of their diabetes self-management practice. Almost all the respondents had knowledge about dietary practices and believed that engaging in good eating habits had beneficial effects on their blood glucose levels. Most participants emphasised “*no or low sugar consumption*” in their meals.

*“Please*, *from the education*, *we’re told not to eat sugar*. *As a result*, *I don’t take sugar*. *Even though I like tea*, *I make raw tea without sugar*.*”* (75-year female respondent)

Furthermore, some of the patients controlled their portion sizes as well as incorporating fruits and vegetables in their diets. One respondent indicated “…*Previously*, *if you are used to eating two balls of banku [*a staple food made from corn dough and cassava dough*]*, *now you must eat one ball only*. *If I was eating four fingers of boiled plantain*, *I should eat two”*

*“I know it’s about my diet*. *I must take lots of vegetable soup*, *‘nkontomire’* [cocoyam leaves sauce] *and garden-eggs”* (added by a 65-year-old female)

Despite the majority who knew about portion size control, there was still some level of confusion regarding the dietary recommendations or the patients considered the alternative to be dear.

*“With respect to the food aspect; getting the recommended food has been a problem*. *I have been advised to eat plantain [and not cassava because cassava has lots of starch] as my main food which I find it difficult to eat most times”* (82-year-old female)

#### Exercise

Exercise was one of the least mentioned self-management practices undertaken by the respondents. Only two out of the 20 participants replied engaging in any form of exercise as part of their diabetes self-management or having knowledge of the benefits of exercising. Some narrated walking long distances to their farmlands that keeps them physically active.

*“I have been told to engage in exercises*. *When you exercise in the mornings*, *it helps reduce your high blood sugar levels*.*”* (a 49-year-old male respondent)

#### Adherence to medication

Most patients said they were compliant with their medications, as well as visiting the clinic regularly for their repeat prescriptions.

*“I take my medicine regularly and attend review appointments a”* (80-year-old female)

#### Foot care practices

More than half were limited in their knowledge in diabetes foot care practices. Some patients indicated that they had not receive any education on foot care practices.

*“We discuss about the dietary management of the disease but not about foot care practices”***(**46-year-old male farmer**)**.,

The few who had received some information reported inspecting their feet occasionally or wearing appropriate footwear to protect themselves, especially low-heeled shoes to prevent wounds from accidents.

*“The previous staff mentioned that for patients with diabetes*, *we should moisturise our feet by applying shea butter on our legs and feet*, *if possible*. *I also realised that for people with diabetes*, *wearing a high-heel can cause you to fall*. *Therefore*, *it is better to wear a low-level shoe”* (59-year-old female)

Furthermore, some of the participants who were farmers were conversant with measures to protect themselves from developing a cut or sore at their workplace.

*“I wear protective clothing like socks and long trousers before putting on shoe to protect my foot from risk of injury whilst working on the farm”* (63-year female farmer)

Some of the patients, however, engaged in behaviours that put them further at risk for foot complications. For instance, some patients said they treated their leg wounds at home.

*“If I get hurt deeply*, *I will report to the facility*. *However*, *with minor sores*, *I manage them myself at home”* (67-year-old female with a one-year history of diabetes)

Other patients, while attempting to engage in healthy behaviours, rather put themselves at risks of injury.

*“Sometimes I do not put on footwear*. *I learnt that when I walk on gravels with my barefoot*, *it provides some form of healing and improved circulation*.*”* (82 years with 10 years duration of diabetes)

### Experiences with diabetes foot assessment

When asked if they have undergone any form of diabetes foot assessment, the vast majority (about 90%) of the respondents reported never having their feet checked as part of their routine management plan. A few who had a previous foot ulcer or amputation shared experiences of having their feet examined for treatment purposes whilst on admission. Some patients otherwise noted that their care providers were willing to attend to them if they had any problems with their feet.

*“No*, *my foot has not been assessed before*. *However*, *we are being told to report any foot problems or ulcers when we notice them*.*”* (65-year-old male with 7 years duration of diabetes)

## Discussion

This study assessed the knowledge and experiences of adults with diabetes on diabetes complications and self- management practices with emphasis on foot care in two Ghanaian district hospitals.

### Knowledge of diabetes and its related complications

This study showed that the majority of patients had some form of knowledge about diabetes and its complications, but their knowledge of the latter was limited. This is similar to a study by Obirikorang et al [12] at Sampa, Ghana which found that 60 percent of the patients with diabetes had no knowledge, 26.9% had inadequate knowledge and only 13.1% had sufficient knowledge about diabetes complications. Stroke and hypertension were the commonly known complications of diabetes among the sample [12]. This is likely because most of the participants in this study also had hypertension, as found elsewhere [20] and both are common non-communicable diseases in Ghana [21]. Foot complications were the least mentioned consequence of diabetes in this study. This contradicts the findings by Obirikorang et al [12] where a slight majority of the participants knew about diabetic foot as the common complication followed by hypertension [12]. Their approach, which involved a closed-ended structured questionnaire, differs from the semi-structured face-to-face interview employed in this study, where patients were asked open-ended questions on diabetes complications, and their responses were followed up with probes that allowed them to share their views and experiences freely and more extensively. However, the results in this study are similar to a descriptive study results in India where only 10 percent of the sample knew diabetes foot problems as a complication of diabetes [22]. The causal pathways for diabetes foot ulcers is well known and include peripheral neuropathy, peripheral vascular diseases, foot deformity or trauma [23]. Unfortunately, our observations during the interviews revealed that participants in this study who had some form of neuropathy were unaware of its connection with foot ulceration, as reported elsewhere [24]. Excluding those with personal experience with foot ulcer or a previous amputation, most participants in this study were not aware of diabetes foot ulcer complication and its associated burden.

Most of the patients said they learned about diabetes and its complications from personal experience or their healthcare providers. They acknowledged the education they received from their care providers and paid attention to them. Given that most of the participants had no formal education, their knowledge of diabetes was therefore testament to the work of healthcare professionals. It is known from experiential learning theory that people who learn from their experiences acquire knowledge from the transformation of those experiences, and the more people are exposed to education, the more they learn from it [25]. This suggests that frequent and effective interactions between healthcare providers and their clients may help increase their knowledge and practices. Our interactions with the healthcare professionals at the diabetes clinics, however, revealed that they were general practitioners and who did not have any specialist training in diabetes care, and patients visited at least monthly for their repeat prescriptions with no other opportunity in between to remain in touch. This suggests a missed opportunity to provide easy regular access for patients, for example to via virtual platforms or telephone, to answer questions patients may have with their disease. Providing specialist training for nurses may also help increase their knowledge in diabetes care, management strategies and knowledge about effective teaching strategies for diabetes care.

### Diabetes self-management practices

Generally, all-inclusive diabetes self-management practices among the patients were uncommon. Most of them relied on their medications and tried to adhere to the dietary recommendations to manage their condition. They paid less attention to self-care practices like foot care due to lack of education and support from their care providers. Some said they never had their feet examined by their healthcare providers.

Nutrition therapy is fundamental in the management of diabetes [26]. Participants’ responses indicated that majority were conversant with reduced intake of sugary foods and controlled portion sizes of food in managing their diabetes. Misconceptions regarding the intake of specific starchy foods like plantains to manage diabetes were present among some of the participants, which in fact, deviate from the dietary recommendation for an individualised meal plan instead of a one-size-fits-all eating pattern [26]. This is also testament to the lack of an adequate nutritional guideline tailored for people with diabetes in Ghana. Further identified factors to non-dietary adherence which corroborates with findings from previous works include: determining the appropriate portion food sizes, lack of knowledge about carbohydrate sources and glycaemic index, cost of food, and family food traditions [13, 27].

Exercise is essential in the prevention and management of Type 2 Diabetes by regulating blood glucose, in addition to positively affecting blood pressure, lipids, cardiovascular incidents, mortality, and quality of life [28]. The majority of the participants in the current study had a generally active lifestyle and occupation. This active lifestyle is typical in most rural African communities [29]. Physical activity thus appeared a component of less concern in managing their diabetes. However, old age and retirement can affect peoples’ capacity to remain active overtime [30]. As a result, patients must be reminded about the benefits of remaining active always and guided on engaging in alternative forms of exercises.

It was striking to find that foot self-care practices were the least acknowledged and practised self-care activity in the setting, especially given that farming, the predominant occupation, put them at risk for foot ulcers. Only a quarter of the participants with an ulcer or history of ulcer or amputation were aware of engaging in daily foot care practices. Unfortunately, certain foot care practices believed to be helpful by some of the participants including walking barefooted and self-treatment of minor wounds, put them at increased risk of ulceration [24]. Anecdotal evidence suggests that these beliefs were mostly from non-clinical sources like radio, peers and relations. While mass media, including radio, is a good source of information, it may be important that personnel who are invited to speak on health topics have locus and expertise to ensure the information shared is evidence based and consistent with national guidelines. It would also be helpful for patients to have reliable contacts with community health personnel to validate any concerns they may have. There is a need to document foot care and other self-management beliefs and practices held by patients with diabetes, and the sources of these information in order to develop effective community level interventions to solving them.

Some patients cited receiving minimal foot care instructions compared to dietary advice from their healthcare professionals, which may account for their poor foot care knowledge. It is largely known that an individualised foot care education coupled with comprehensive diabetes foot assessment for patients with diabetes lead to improved knowledge, self-protective behaviour, and reduction of foot complications [31]. Accordingly, healthcare providers should receive continuous training to improve their skills and knowledge to support their patients in that regard [32]. The literature describes foot care activities to include: daily feet inspection, annual diabetes foot examination by a professional, foot hygiene, seeking early professional care for sores or cuts, appropriate footwear, not walking barefoot, and not removing ingrown toenails, calluses or corns by oneself [33]. Though foot care practices may not preclude the risk of developing foot ulcers, high ulcer risks have been reported in patients that do not engage in such practices [34].

### Diabetes foot assessment

Diabetes foot assessment refers to examinations to identify individuals at high risk for diabetes-related foot conditions. It includes taking a detailed medical history, screening for foot complications, reviewing appropriate footwear, education on foot care, providing early treatment and referral for specialist foot care when necessary [35]. In both hospitals, patients indicated there were no scheduled foot assessment plans for them, and nearly all, except for those with previous ulcer or amputation have not previously undergone a diabetes foot assessment by a healthcare professional. The probable explanation could be the lack of standardised or national diabetes foot care and prevention guideline in Ghana. This does not correspond to standard international guidelines which recommends performing at least an annual foot examination for people with diabetes [5]. Others may include limited skilled personnel and the lack of resources to conduct foot examinations in most diabetes care centres in the country. Developing a national diabetes care plan for healthcare professionals, especially nurses and community care nurses, and the provision of specialist training will ensure adequate knowledge and improved patient care.

## Conclusion

This study showed that patients with diabetes were knowledgeable about the disease but not so much so about the complications. Most of the participants received information from their care providers or learned from their own experiences. The education received on diabetes-self management practices focused much on medication and dietary aspects but less on foot care activities. Hence, some of the patients rarely participated in daily foot care practices. Also, there were no scheduled diabetes foot examination plans for patients, and majority have never undergone a foot examination with a professional, except for those who have developed active foot complications. Given the rural and urban inequalities in healthcare in Ghana, this study suggests that the situation in rural areas may be dire. Undertaking early footcare practices and supporting patients to do same is vital to early detection and reduction of disabilities associated with foot ulcers. Healthcare professional such as diabetes educators, nurses, physicians and podiatrists play vital roles in improving patient’s metabolic control and prevention of diabetes complications among patients by providing appropriate education and treatment to them.

## Implications for clinical practice

The current study reveals that patients with diabetes in the study districts have limited knowledge about complications and have different perceptions regarding diabetes self-management practices. Therefore, it will be beneficial to increase diabetes awareness campaigns and encourage self-management practices through mass media and education campaigns. Given the importance of self-management to glycaemic control, this also emphasises the need for community approaches in the delivery of diabetes care. This may require involvement of community health nurses to follow up and monitor patients on how they manage their condition at home. Diabetes self-management education programmes should be delivered in a tailored, organised and frequent manner, with a demonstration of skills that correspond to the level of knowledge of targeted individuals [32]. The government through its Ministry of Health must establish policies that will implement effective diabetes management national guidelines particularly, annual foot checks for patients with diabetes in the country. Healthcare professionals in diabetes care should receive specialist training to deliver person-centred diabetes care as well as effective consultation skills for people with diabetes. More specialist podiatrists should be trained across the different parts of the country to assess, manage, and treat foot complications especially for patients with diabetes.

## Strengths and limitations

This is an important study that contributes to the growing literature on diabetes self-management, and especially on foot care practices in a predominantly urban setting in Ghana. It is among the first to use a phenomenological design to provide in-depth understanding of patients’ knowledge of diabetes and detailed experience of self-care practices, with focus on footcare. Previous studies have used quantitative approaches which largely miss detailed patient experiences and narratives or considered other aspects of self-management.

This study would have benefited more from the views of healthcare professionals involved in the care of diabetes to connect with the patients’ perspectives explored. Therefore, future studies should examine both sides for better comparable results. This study did not assess self-monitoring of blood glucose as part of diabetes self-care practices. In Ghana and elsewhere in sub-Saharan Africa, most individuals with diabetes lack personal glucose monitoring devices in their homes for self-monitoring. Therefore, monitoring of blood glucose occurs only during routine appointment at the diabetes clinic and periods of patients’ illnesses [36]. Future studies should explore how people with diabetes self-monitor their blood glucose levels, especially in the community setting. Finally, we advise that the findings in this study be carefully applied given that our study was a qualitative study conducted in only two urban public hospitals in Ghana with limited number of participants. Further studies in other healthcare settings- private clinics, regional hospitals or other primary care facilities- will be required to corroborate our findings.

## Supporting information

S1 File Patient interview guide for diabetes management and foot care practices in Ghana. (DOCX)

## List of abbreviations

ADA: American Diabetes Association
DSM: Diabetes self-management
IDF: International Diabetes Federation
LMIC: Low and middle-income countries
WHO: World Health Organization
