# Supplementary material for: Patients’ knowledge of diabetes foot complications and self-management practices in Ghana: A phenomenological study
Source: PLoS One. 2021 Aug 25;16(8):e0256417. doi: 10.1371/journal.pone.0256417 (PMC8386847; doi:10.1371/journal.pone.0256417)
Supplement: S1 File — (DOCX) [file pone.0256417.s001.docx]

## S1 File. Patient interview guide for diabetes management and foot care practices in Ghana.

**PART I**Patient demographic and vital statistics

1. Age (in years) …………
2. Sex
   1. Male …..
   2. Female …….
3. Body mass index
4. Blood pressure (Indicate BP at current clinic) ……………………….
5. Fasting blood glucose (Indicate the latest FBG level)……………………..
6. Educational status (Indicate patients highest level of education) ………………..
7. Occupation ……………………………….
8. Past medical history (List any co-morbidities)
9. Duration of diabetes (State number of years)
10. History of diabetes foot ulcer (Yes/No)
    1. No history of ulceration …………..
    2. Previous ulceration ………..
    3. Active ulcer …………..
    4. Amputation ………...

**Part II**

1. What do you know about diabetes?
2. Could you tell me about your experience with diabetes?
3. What do you think are the important aspects to consider in your diabetes management?
4. What do you think are some of the consequences if your blood sugar level is not well controlled? Or could you tell me about diabetes complications you know

(*If patient mentions foot ulcer or amputation-go ahead to ask if he/she knows anyone who has had an amputation or his/her knowledge about foot ulcer because of diabetes*)

1. What do you think is the cause of foot problems in people with diabetes or cause of amputation in patients with diabetes?
2. Do you think patients who have diabetes should have any special concerns for their feet?
3. Have you received any education regarding how to care for your feet before? (*If yes, find out where they received the information and who taught you?*)
4. Do you examine your feet as part of managing your diabetes?
5. Have your feet been checked at a diabetes clinic before as part of your routine assessment?
6. Have you spoken to a clinician (nurse or doctor) about your feet before? (*If Yes, find out why*)
7. Do you experience any abnormal sensations on your feet?
8. Do you think these symptoms put you at risk for an ulcer?
9. Do you feel you have enough information that will allow you to care for your feet on your own?
10. If you developed a developed a small wound on your feet, what would be your reaction to it? Where will you seek treatment from?
11. What do you think are some of the things you can do to avoid foot problems?
